# Alternative Molecular Methods for Improved Detection of Meningococcal Carriage and Measurement of Bacterial Density

**DOI:** 10.1128/JCM.01428-16

**Published:** 2016-10-24

**Authors:** Olivier Manigart, Jacinta Okeakpu, Aderonke Odutola, Sheikh Jarju, Ebenezer Foster-Nyarko, Kanny Diallo, Anna Roca, Beate Kampmann, Umberto D'Alessandro, Samba Sow, Martin Antonio, Martin J. Maiden, Ray Borrow, James M. Stuart, Caroline L. Trotter, Brian M. Greenwood

**Affiliations:** a Faculty of Infectious and Tropical Diseases, London School of Hygiene & Tropical Medicine, London, United Kingdom; b Medical Research Council Unit, Fajara, The Gambia; c Centre de Développement des Vaccins, Bamako, Mali; d Department of Zoology, University of Oxford, Oxford, United Kingdom; e Faculty of Epidemiology and Population Health, London School of Hygiene & Tropical Medicine, London, United Kingdom; f Department of Paediatrics, Imperial College, London, United Kingdom; g Institute of Tropical Medicine, Antwerp, Belgium; h Vaccine Evaluation Unit, Public Health England, Manchester, United Kingdom; i Department of Veterinary Medicine, University of Cambridge, Cambridge, United Kingdom; Brigham and Women's Hospital

## Abstract

Conventional methods for detecting pharyngeal carriage of Neisseria meningitidis are complex. There is a need for simpler methods with improved performance. We have investigated two alternative approaches. Three pharyngeal swabs were collected from 999 pupils aged 10 to 18 years in The Gambia. Carriage of N. meningitidis was investigated by using three different methods: (i) plating on Thayer-Martin selective medium and testing by conventional microbiological methods followed by PCR testing; (ii) seeding in Todd-Hewitt broth (THB) and, after culture overnight, testing by PCR; and (iii) compression of the swab on filter paper and, after DNA concentration, testing by PCR. PCR after culture in THB was more than twice as sensitive as conventional methods in detecting N. meningitidis (13.2% versus 5.7%; *P* < 0.0001). PCR after DNA extraction from filter paper had a sensitivity similar to that of conventional methods (4.9% versus 5.7%; *P* = 0.33). Capsular genogroups detected by broth culture were genogroups W (21 isolates), B (12 isolates), Y (8 isolates), E (3 isolates), and X (2 isolates), and 68 meningococci had the capsule-null intergenic region. The distributions of genogroups and of capsule-null organisms were similar with each of the three methods. The carriage density in samples extracted from filter paper ranged from 1 to 25,000 DNA copies. PCR of broth cultures grown overnight doubled the yield of N. meningitidis carriage isolates compared with conventional methods. This approach could improve the efficiency of carriage studies. Collection on filter paper followed by quantitative PCR could be useful for density measurement and for carriage studies in areas with limited resources.

## INTRODUCTION

Infection with Neisseria meningitidis is usually characterized by asymptomatic, or minimally symptomatic, carriage of bacteria in the pharynx; meningococcal septicemia and/or meningitis is a rare event that occurs in as few as 1/100 to 1/1,000 colonized individuals. For this reason, selective pressure induced by antimicrobials or by naturally acquired or vaccine-induced immunity is exerted primarily on bacteria carried by asymptomatic carriers. Therefore, the epidemiology of meningococcal infections cannot be understood fully without considering carriers (1, 2).

Carriage of N. meningitidis is relatively uncommon, and thus determining carriage prevalence requires large-scale surveys (3), which are usually conducted by using microbiological techniques developed several decades ago. Detection of meningococcal carriage involves three main steps that could be improved, namely, sample collection, transport to the laboratory, and identification of meningococci. Most studies have focused on the optimization of methods for collecting pharyngeal swabs; these studies have shown that a peroral swab taken behind the uvula is most likely to yield a positive result and that taking two sequential swabs increases sensitivity (4–6). Until recently, little has been done to improve methods of transport (7, 8), and we are aware of only two studies aimed at improving the identification of meningococci by using molecular methods (9, 10). PCR testing on DNA extracted directly from a swab has been employed in some studies, but a recent investigation of UK adolescents showed that this method was less sensitive than conventional culture followed by PCR (11). We have developed two new methods for detecting meningococcal carriage, molecular analysis of extracted DNA obtained either after culture overnight in Todd-Hewitt broth (THB) or from a filter paper on which a swab had been pressed, and compared the results obtained by these methods to those obtained by conventional microbiology and confirmed by PCR in a study of meningococcal carriage in 999 Gambian schoolchildren.

## MATERIALS AND METHODS

### Study design and participants.

**FIG 1:**
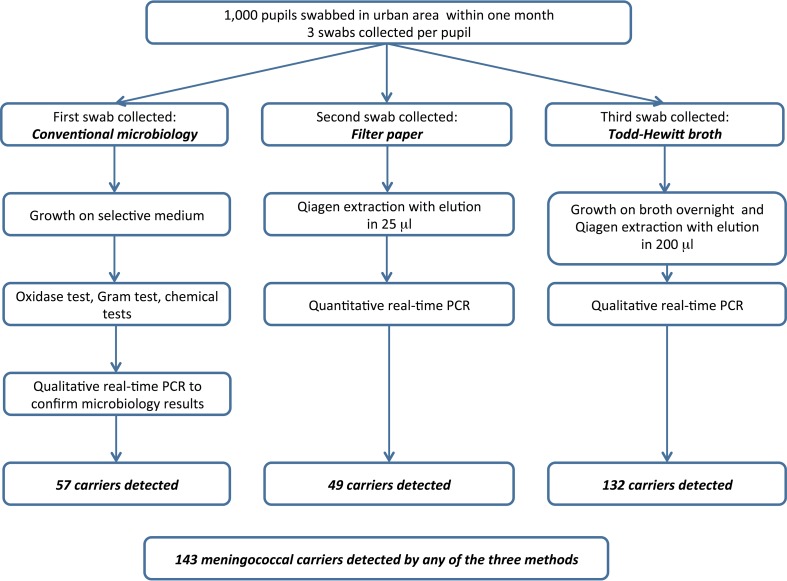
Summary of the methodology employed in the study.

Following sensitization of the community, and with permission from the educational authorities, a cross-sectional carriage survey was undertaken from 1 to 30 July 2013, the start of the rainy season, in children attending middle or high schools in the periurban area of Fajikunda, The Gambia, West Africa. Healthy school attendees aged between 10 and 18 years were recruited sequentially until 1,000 had been enrolled. No children or parents invited to join the study refused to participate. Written, informed consent was obtained from 18-year-old students. Assent and written consent from a parent or guardian were obtained from students aged 10 to 17 years. A questionnaire that investigated potential risk factors for meningococcal carriage was administered to all participants. Three pharyngeal swabs were then collected from each student on the same occasion. One swab was streaked directly onto a Thayer-Martin selective agar plate, a second was placed into THB, and a third was smeared onto a filter paper strip. Children were randomized prior to collection of the samples to one of three groups, which indicated the order in which the three samples were to be collected (Fig. 1), to ensure that the first, second, and third swabs had an equal chance of being tested by each of the three laboratory methods.

The study was approved by the Scientific Coordinating Committee of the Medical Research Council (MRC) Unit, The Gambia; by the Gambian Government/MRC Joint Ethics Committee; and by the Ethical Committee of the London School of Hygiene & Tropical Medicine.

### Conventional microbiology.

The conventional methods employed to identify N. meningitidis by the MenAfriCar Consortium were described in detail previously (12) and are reported briefly here. Swabs were streaked directly onto a modified selective Thayer-Martin agar plate in the field, and plates were held in a 5% CO_2_ jar until they were transported to the laboratory within 6 h of collection. After 24 h of subculture on chocolate agar plates, an oxidase test and a Gram stain were performed. All oxidase-positive, Gram-negative diplococci (OPGNDC) were tested for β-galactosidase activity with *ortho*-nitrophenyl-β-d-galactopyranoside (ONPG), for γ-glutamyltransferase (GGT) activity, and for butyrate esterase (tributyrin) activity. ONPG-negative, GGT-positive, tributyrin-negative bacteria were serogrouped by slide agglutination, initially with serogroup A and W antisera and then, if negative, with serogroup X and Y antisera. DNA was extracted from all OPGNDC isolates by extraction with a Qiagen kit of a bacterial suspension boiled for 20 min and then tested by multiplex real-time PCR (RT-PCR) as described below. In the conventional microbiology group, OPGNDC isolates that were ONPG negative, GGT positive, and tributyrin negative and also PCR positive (see below) were considered to be N. meningitidis isolates.

### Broth culture.

Prior to the field study, the ability of two broth cultures to support the growth of N. meningitidis was tested by using aliquots spiked with serial dilutions of a serogroup A reference strain (ATCC 13077), starting with a dilution of ∼1,200 × 10^8^ CFU per ml. The first medium tested was Mueller-Hinton broth (MHB; Oxoid, Basingstoke, UK) supplemented with VCNT (containing vancomycin [3 mg/liter], colistin [7.5 mg/liter], nystatin [1,250 U/liter], and trimethoprim [5 mg/liter]) (catalog number SR0091E; Oxoid). The second medium investigated was THB (Oxoid, Basingstoke, UK) supplemented with 0.5% yeast, rabbit serum (B&K Universal Ltd., Grimston, East Yorkshire, UK) to facilitate preincubation before arrival at the laboratory, and the same antibiotic combination as the one described above. DNA was extracted from broth cultures by using a Qiagen kit according to the manufacturer's instructions (with elution in 200 μl). Todd-Hewitt medium supported bacterial growth at higher dilutions than did Mueller-Hinton broth (Table 1), and this medium was used in the field study.

**TABLE 1:** Comparative efficacies of Todd-Hewitt and Mueller-Hinton broths in supporting growth of N. meningitidis cultures overnight^*a*^

Avg log_10_ no. of genome copies by spectrophotometry	Avg Todd-Hewitt broth *porA C_T_*	Avg Mueller-Hinton broth *porA C_T_*
7.47	11.28	14.38
6.48	13.83	16.73
5.57	18.08	22.45
4.41	20.10	24.03
3.57	16.38	21.02
2.71	20.60	25.85
1.85	25.09	29.51
0.90	27.95	33.33
∼1	35.21	Undetermined
None	Undetermined	Undetermined

a PCR positivity obtained with serial dilutions of a serogroup A reference strain of N. meningitidis is shown.

### Filter paper cards.

The ability of filter paper strips (FTA MiniCard, catalog number WB 120055; Whatman) to preserve N. meningitidis DNA prior to DNA extraction was explored in the laboratory by using serial dilutions of a suspension of a serogroup A meningococcal reference strain (ATCC 13077); samples spiked with different dilutions of the bacterial suspension were spotted onto filter paper strips and held at room temperature (18°C to 23°C) for 48 h prior to DNA extractions using a Qiagen kit. Next, elution was done twice with a volume of 25 μl, a lower volume than the usual 200-μl volume, in order to concentrate DNA. In the laboratory, it was possible to detect ∼1 N. meningitidis *porA* gene copy by RT-PCR. In the field, a swab was smeared directly onto a MiniCard, which was held at room temperature for several weeks prior to extraction. In the field, a swab was smeared directly onto a MiniCard, which was held at room temperature for 6 to 12 months prior to extraction, as it is well known that DNA collected onto FTA cards can be preserved for years at room temperature. By using a punch, a small segment of the filter paper (∼6 mm in diameter) was obtained from the center of the smear, and DNA was extracted as described above. A second sample was obtained as close as possible to the center and tested by using the same procedure, as it was considered that bacteria might have been concentrated off-center when the swab was compressed onto the MiniCard.

### Multiplex RT-PCR for N. meningitidis detection.

Detection of N. meningitidis was undertaken by targeting both the *porA* and *sodC* genes and the capsule-null intergenic region (*cnl*) simultaneously. Genogrouping of all *porA*- and/or *sodC*-positive samples, considered N. meningitidis, was done according to a method developed previously by Wang et al. (13) for genogroups A, W, and Y, followed by genogroups B, C, and X. All samples of N. meningitidis that could not be characterized in this way underwent a multiplex PCR for genogroups H, E, and Z (see Table S1 in the supplemental material). The cycling conditions were the same for all tests: 1 cycle of 2 min at 50°C, 1 cycle of 10 min at 95°C, and 50 cycles of 15 s at 95°C and 1 min at 60°C. The ABI 7500 fast cycler was used to perform the reaction, and the results were analyzed by using 7500 fast software. Samples were kept at 4°C after amplification. For both the examination of THB samples and PCR confirmation of isolates obtained by conventional microbiological methods, a stringent threshold cycle (*C_T_*) value of 25 was used for both the *porA* and *sodC* genes to select true positives. This threshold was shown to be optimal by comparison with positive controls diluted at different concentrations and plotted on standard curves. For genogrouping multiplex PCR, the conventional *C_T_* of 35 was used as the threshold for positivity, and samples with values of between 36 and 40 were retested after 10-fold dilution according to the method described previously by Wang et al. (13). For filter paper testing, using positive controls at different dilutions plotted on standard curves, better results were obtained with the *porA* monoplex RT-PCR than with the multiplex *porA-sodC-cnl* assay during our preliminary evaluation, and therefore, we used only *porA* monoplex RT-PCR during the field study, with a *C_T_* threshold of 35 being the criterion of positivity.

### Measurement of bacterial density.

For calculation of bacterial density, N. meningitidis genogroup W reference strain ATCC 35559 was harvested from a culture grown overnight and diluted in phosphate-buffered saline (PBS) to reach a 4.0 McFarland standard. Serial dilutions were made, and DNA extraction was done by using Qiagen kits according to the manufacturer's recommendations to build standard curves for DNA quantification by RT-PCR. Standard curves of DNA measurements were made by using a NanoDrop spectrophotometer (Thermo Scientific, USA) and a PicoGreen double-stranded DNA (dsDNA) quantitation assay (Life Technologies, France). The average of both DNA measurements was used when values were discrepant. Genome copy numbers were estimated by using the formula mass = number of DNA base pairs per genome × 1 mol/6.022 140 × 10^23^ × 660 g per mol, where 6.022 140 × 10^23^ is Avogadro's number (molecules per mole) and 660 g/mol is the average molecular weight of a double-stranded DNA molecule. Extracted bacterial DNA was then subjected to PCR as described above.

### Statistical analyses.

The study was designed to have 80% power to detect an increase in carriage prevalence from an estimated carriage prevalence of 5% in the conventional microbiology group to at least 7% from paired samples by employing one of the two novel approaches to carriage detection; this required a sample size of 870. Data were analyzed by using Stata v12.0. Sensitivities were compared between methods by using the exact McNemar significance probability. Differences in carriage prevalence by group (e.g., age and sex) were investigated by using chi-squared tests and logistic regression.

## RESULTS

One thousand students were recruited into the study; 1 child was excluded because of a missing questionnaire, leaving 999 for analysis. The majority of students (*n* = 859) were aged 10 to 13 years, and the remainder (*n* = 138) were 14 to 18 years old; age was not recorded for 2 children. More male than female students were enrolled (589 versus 410). Pharyngeal carriage of N. meningitidis was detected in 143 students by one method or the other (overall carriage prevalence, 14.3%). Carriage prevalences were similar in males (80/589 [15.3%]) and females (63/410 [13.5%]) (*P* = 0.428). Carriage was not associated with age within the limited age range investigated (*P* = 0.519).

One hundred eleven oxidase-positive, Gram-negative diplococci were isolated by using the conventional microbiology approach; 49 were characterized as N. meningitidis by biochemical methods (ONPG, GGT, and tributyrin tests). This identification was confirmed by multiplex PCR for all but three samples. An additional 21 OPGNDC isolates had a weak reaction with one of the three biochemical tests and were tested by RT-PCR. Eleven isolates were positive for N. meningitidis upon RT-PCR testing, making a total of 57 N. meningitidis isolates detected by conventional microbiology followed by RT-PCR (carriage prevalence, 5.7%). Several false-positive reactions were seen by using seroagglutination. For example, 13 N. meningitidis genogroup A isolates were identified by seroagglutination, which were not confirmed by N. meningitidis genogroup A-specific genogrouping RT-PCR. Genogrouping of the 57 N. meningitidis isolates identified the following genogroups in decreasing order of prevalence: *cnl* (21 isolates), genogroup W (11 isolates), genogroup B (5 isolates), genogroup Y (3 isolates), genogroup E (2 isolates), genogroup C (2 isolates), and dual *cnl*-genogroup W (2 isolates) and *cnl*-genogroup Y (1 isolates). Ten samples could not be classified through genogrouping PCR (nongroupable [NG]). All samples that were *sodC* positive and *porA* negative were *cnl* or NG isolates.

**FIG 2:**
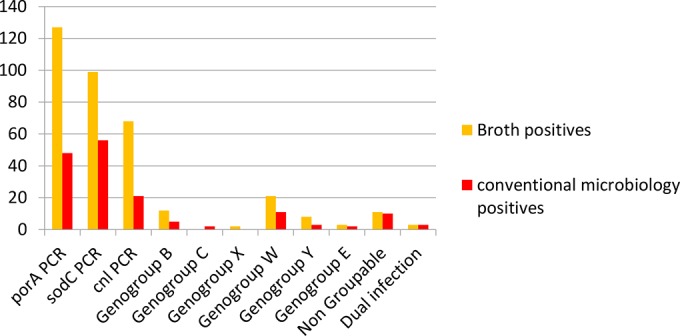
Comparison of N. meningitidis positivity using *sodC* and *porA* and genogroup characterization by real-time PCR on isolates after conventional microbiology versus culture overnight in Todd-Hewitt broth.

Culture in THB overnight followed by RT-PCR identified 132 carriers of N. meningitidis (carriage prevalence, 13.2%), a marked increase in sensitivity compared to that of the conventional method (*P* < 0.0001). Sixty-eight of the 132 N. meningitidis isolates (51.5%) possessed only the *cnl* intergenic region (Fig. 2). Genogroups detected following broth culture were genogroups W (isolates 21), B (12 isolates), Y (8 isolates), E (3 isolates), and X (2 isolates). Four additional samples (not classified as meningococci in the primary analyses) had doubtful results, with a *C_T_* of between 25 and 30. No genogroup A, C, H, or Z N. meningitidis isolates were detected. Eleven strains grown in THB were NG isolates, and 7 dual carriers were identified. Overall, the genogrouping results of the two methods matched well, but there were a few discrepancies between the results obtained with THB culture and those obtained by the conventional method: two genogroup C and two genogroup E isolates were not confirmed by THB culture. The superiority of THB culture over conventional microbiology in detecting both *cnl* strains and different genogroups of meningococci is demonstrated in Fig. 2.

**FIG 3:**
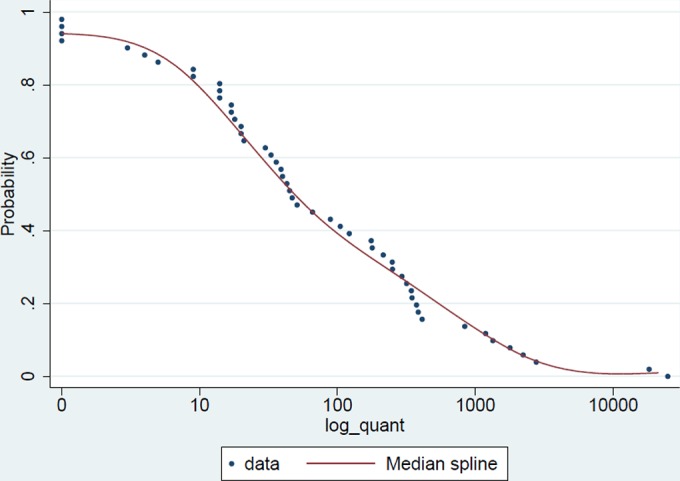
Bacterial density distribution measured by the number of copies of a fragment of the *porA* DNA gene.

Forty-nine N. meningitidis carriers (carriage prevalence, 4.9%) were identified by using filter paper MiniCards, a prevalence similar to that using the conventional method (*P* = 0.33). Among these carriers, 18 carried the *cnl* gene with *C_T_* values of ≤35. Genogrouping RT-PCR could not be performed on the other samples due to the low volume of the eluant used to concentrate the DNA for N. meningitidis detection and the fact that repeated tests were done to verify positive tests. The density of carriage, as reflected in the number of DNA copies obtained from the filter paper, varied between 1 and 25,000 DNA copies, with a median value of 59 copies (Fig. 3). There was no association between a subject's age and carriage density. Additional testing with a second punch taken from the center of the smear allowed the identification of one additional carrier. The median density from 13 second-punch samples (2.0 [95% confidence interval {CI}, 1.0, 10.8]) was lower than that found in 48 first-punch samples (58.5 [95% CI, 35.0, 226.0]), emphasizing the importance of taking a punch from the center of the smear. Only 2 of 49 subjects had >10,000 copies of *porA* DNA, and only 7 had >1,000 copies.

**FIG 4:**
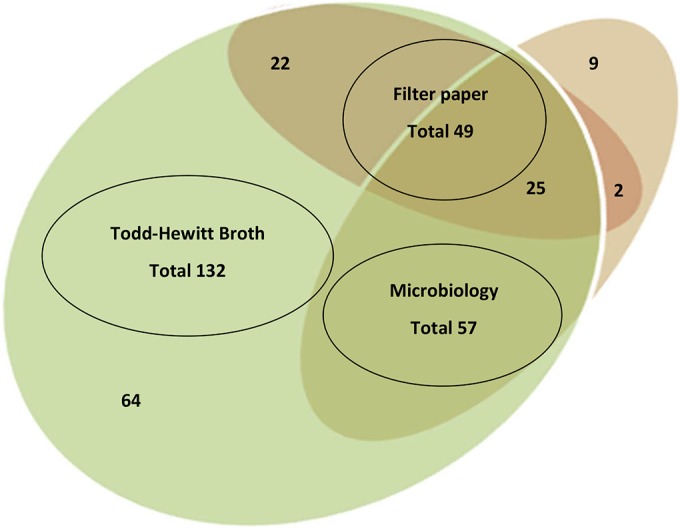
Venn diagram representing positive swabs collected and tested by the following three methods: (i) conventional microbiology and PCR after DNA extraction, (ii) collection on filter paper and direct PCR after DNA extraction, and (iii) culture overnight in Todd-Hewitt broth and direct PCR after DNA extraction.

The overlap in the detection of carriage between the different methods is shown in a Venn diagram (Fig. 4). Most carriers identified by conventional microbiology (80.7%) were also identified by broth culture, and 47.4% were identified by using filter paper. Forty-seven of the 49 carriers identified by using filter paper were also identified by using THB culture, and 27 were identified by using conventional microbiology. By using the *porA* and/or *sodC* gene to define positivity, and assuming that detection by any method was a true positive, the sensitivity of conventional microbiology was 39.6%, that of the filter paper method was 34% (using a *porA* monoplex RT-PCR), and that of THB culture was 91.6%.

## DISCUSSION

Detection of meningococcal carriage in children attending schools in Fajikunda, The Gambia, was enhanced >2-fold compared to conventional culture by culture overnight in a selective medium and subsequent detection of meningococcal DNA. The genogroup distribution of carriers detected by using broth culture was similar to that seen with conventional culture, suggesting that culture overnight did not have a major selective effect, although genogrouping of a few strains did not match between methods. The enhanced sensitivity of the broth culture approach probably reflects the fact that many carriers have only a low density of bacteria on the surface of the pharyngeal mucosa and the fact that liquid broth provides N. meningitidis a better environment for immediate growth than solid Thayer-Martin medium. The broth culture technique is cheap and easily implementable, with collected samples being cultured overnight, boiled in PBS, and then stored at −20°C for later RT-PCR analysis at a convenient time.

A limitation of this study is that the preservative medium made of skimmed milk, (tryptone), glucose, and glycerin [S(T)GG medium], commonly used for transport in pneumococcal carriage studies, was not evaluated. However, in a qualitative and semiquantitative comparison of preservation in STGG medium with conventional direct plating in the field, O'Brien et al. demonstrated that direct plating was slightly superior to STGG medium for the recovery of Streptococcus pneumoniae (14). In a recent study of 601 students in Portugal (10), a similar prevalence of meningococcal carriage was obtained by culture and by direct *sodC* RT-PCR on samples collected in STGG medium (13.3% and 14.5%, respectively) (Adam Finn, personal communication). Our study demonstrated that broth can be used as both transport and culture media, but more studies are needed to define which medium is optimal. The use of rabbit serum is a limitation of our method, as this reagent has to be shipped frozen, a challenge for resource-limited countries. Further studies are needed to define whether the efficiency of detection is similar without this reagent and whether the efficacy of broths for short-term culture of N. meningitidis can be improved further.

Collection of blood spots on filter paper strips with subsequent DNA extraction and molecular analysis is a technique used widely in studies of other infectious diseases, including malaria and HIV (15), but, as far as we are aware, the filter paper technique has not been used for the collection of meningococcal DNA. We tested filter paper eluates on which a swab had been pressed by *porA* monoplex RT-PCR only rather than by *porA* and *sodC* RT-PCR, as this gave us better results during the evaluation of spiked samples, so the comparison of results obtained with the filter paper technique and conventional microbiology reflects a comparison of two optimum techniques rather than a comparison of directly similar PCR methods. Employing the optimum techniques for each method, the filter paper approach had a sensitivity similar to that of the conventional method. However, it has the advantage that samples can be stored at room temperature for prolonged periods and readily transported to a central laboratory without degradation of DNA prior to analysis, avoiding the need for a cold chain or transport medium with temperature monitoring. Another advantage of the filter paper technique is that it does not involve any culture preamplification and thus allows direct measurement of the density of bacteria present on the swab and, hence, an indication of the density of pharyngeal carriage in an individual. We found a wide range of bacterial densities in the filter paper samples, with a density distribution similar to that found in UK students (16). Since high-density carriers are likely to be more infectious than individuals carrying only a few bacteria, determination of carriage density is likely to become an important endpoint in future meningococcal vaccine trials. Strips were stored for up to 6 months at room temperature before testing, and experience with malaria filter paper blood spots suggests that samples could be held for up to 6 years without a loss of DNA (17), especially when short DNA fragments are targeted. The simplicity of the filter paper technique could make this a useful approach when carriage surveys are needed in areas with few facilities or when there is a need to define the groups with the highest bacterial densities; this warrants further investigation.

The increased yield of N. meningitidis detected by using short-term culture in THB needs confirmation but suggests that meningococcal carriage studies to date have considerably underestimated the true level of pharyngeal carriage of N. meningitidis. By using this new technique, the size and cost of carriage studies, for example, those required to evaluate the impact of new meningococcal vaccines, that are being developed could be reduced.

## Supplementary Material

Supplemental material
