# Supplementary material for: Alternative Molecular Methods for Improved Detection of Meningococcal Carriage and Measurement of Bacterial Density
Source: J Clin Microbiol. 2016 Oct 24;54(11):2743–8. doi: 10.1128/JCM.01428-16 (PMC5078552; doi:10.1128/JCM.01428-16)
Supplement: Supplemental material [file supp_54_11_2743__index.html]

Supplemental material 

# Alternative Molecular Methods for Improved Detection of Meningococcal Carriage and Measurement of Bacterial Density

## Supplemental material

- Supplemental file 1 -

  Table S1 (Primers and probes used for real-time PCR)

  PDF, 273K
