# Supplementary material for: Alternative Molecular Methods for Improved Detection of Meningococcal Carriage and Measurement of Bacterial Density
Source: J Clin Microbiol. 2016 Oct 24;54(11):2743–8. doi: 10.1128/JCM.01428-16 (PMC5078552; doi:10.1128/JCM.01428-16)
Supplement: Supplemental material [file JCM.01428-16_zjm999095205so1.pdf]

Supplement

Table S1

Primers and probes used for real-time PCR

Targeted gene (species specific)	primer and probe name	5'-3' nucleotides sequence	dye	probe type
sodC (Nm)	sodC-F	GCA CAC TTA GGT GAT TTA CCT GCA T		
	sodC-R	CCA CCC GTG TGG ATC ATA ATA GA		
	sodC-probe	CAT GAT GGC ACA GCA A	NED	MGB
porA (Nm)	RT_porA-F	GCCGGCGTTGATTATGATTT		
	RT_porA-R	AGTTGCCGATGCCGGTATT		
	RT_porA-P	CTTCCGCCATCGTGTC	FAM	MGB
Cnl	RT_cnl-Fa	GAATTGCATAGGTTATCCAAAATCAC		
	RT_cnl-Fb	GAGTTGCACAGATTATCCAGAATCAC		
	RT_cnl-P	ATAAAACCGGTGCCGCC	VIC	MGB
	RT_cnl-R	TTTGCCCGATACAATCTGAAAG		
A	F2531	AAAATTCAATGGGTATATCACGAAGA		
	R2624	ATATGGTGCAAGCTGGTTTCAATAG		
	RT_csaB_P	CTAAAAGTAGGAAGGGCACTT	VIC	MGB
W	F857	TATTTATGGAAGGCATGGTGTATG		
	R964	TTGCCATTCCAGAAATATCACC		
	Pb907i FAM	AAATATGGAGCGAATGATTACAGTAACTATAATGAA	FAM	BHQ
X	F173	TGTCCCCAACCGTTTATTGG		
	R237	TGCTGCTATCATAGCCGCC		
	Pb196 CY5	TGTTTGCCCACATGAATGGCGG	Cy5	BHQ

B	F737	GCTACCCCATTTCAGATGATTTGT		
	R882	ACCAGCCGAGGGTTTATTTCTAC		
	Pb839i CY5	AAGAGATGGGYAACAACTATGTAATGTCTTTATTT	Cy5	BHQ
C	F478	CCCTGAGTATGCGAAAAAAATT		
	R551	TGCTAATCCCGCCTGAATG		
	Pb4951 FAM	TTTCAATGCTAATGAATACCACCGTTTTTTTGC	FAM	BHQ
Y	F787	TCCGAGCAGGAAATTTATGAGAATAC		
	R929	TTGCTAAAATCATTCGCTCCATAT		
	Pb1099i HEX	TATGGT GTACGATATCCCTATCCTTGCCTATAA	HEX	BHQ
Z	cszC-1_Fwd1	CAGGCCGAAGAGCGTTATCA		
	cszC-1_Rev1	CGCCATTCAGGGCGATT		
	cszC-1_probe1	ACAGCTCTGGCCTTAG	Cy5	MGB
E	RT_cseE-F	GAGGCTGGCAATGACCAATT		
	RT_cseE-R	CCCAGCATATCGACAACCAA		
	RT_cseE-P	ATCTTATGTGAACGTGGCGC	FAM	MGB
H	RT_cshC-F	AAGCCCGTTCCAAGATCATG		
	RT_cshC-R	GCGGTTTGGAGAAATAATATGTGTT		
	RT_cshC-P	AATGTCAGCCGTAACTT	VIC	MGB

6
